# Translating fathers’ support for breastfeeding into practice

**DOI:** 10.1017/S1463423621000682

**Published:** 2021-11-03

**Authors:** Sharin Baldwin, Debra Bick, Alison Spiro

**Affiliations:** 1 Warwick Clinical Trials Unit, University of Warwick, Warwick, UK; 2 Learning and Organisational Development, London North West University Healthcare Trust, London, UK; 3 University Hospitals Coventry and Warwickshire, Warwick, UK; 4 Brunel University, London, UK

**Keywords:** breastfeeding, fathers, health visitors, infant feeding, midwives

## Abstract

Breastfeeding has numerous health benefits for the mother and child. For breastfeeding to be successful and continue for longer, women need adequate support. Fathers/partners play an important role in providing this support to women, but research suggests that fathers/partners often feel inadequately informed and supported by health professionals. Midwives and health visitors are in ideal positions to offer women and their partner’s timely and relevant breastfeeding information and support throughout the perinatal period. This article discusses the benefits of breastfeeding, presents research evidence of the crucial role fathers/partners play in promoting and supporting breastfeeding, and recommends ways in which health professionals can provide breastfeeding information and support to fathers/partners.

## Introduction

Breastfeeding carries a number of health benefits for the mother and child, as well as wider economic benefits to society. The supportive partner of the breastfeeding person has an important role to play in building confidence in families and therefore strategies to increase breastfeeding rates need to include better support for partners/fathers. This requires a shift in training and practice for healthcare professionals, where historically the main focus for breastfeeding promotion and support may have only been on the mother rather than also including the father. This article discusses the benefits of breastfeeding and presents findings from the UK based New Dad Study (Baldwin *et al*., 2018; 2019; 2021) to highlight the crucial role that fathers play in promoting and supporting breastfeeding, and recommends ways in which health professionals can provide breastfeeding information and support to fathers/partners.

## Background

The World Health Organisation (WHO) Global Strategy for infant and Young Child Feeding was first published in 2003 and subsequently reviewed in 2020, in response to concerns about declining global rates of breastfeeding. it stated that only 40% of infants were exclusively breastfed for 6 months globally and estimated that around 820 000 children’s lives could be saved globally, if all children were optimally breastfed for up to 23 months (WHO, 2020). The strategy aimed to revitalise efforts to promote, protect, and support breastfeeding and build on previous initiatives such as the innocenti Declaration (Unicef, 2005) and Baby Friendly Hospital initiative’s Ten Steps to Successful Breastfeeding (WHO, 1991). it called on all governments to develop and implement policies and strategies to promote breastfeeding. it recommended that the support offered to women should be delivered by well-trained staff, able to offer counselling skills, and link with other support agencies in the community.

Evidence shows that breastmilk offers infants optimal nutrition as it is biologically designed for an infant’s gut, easy to digest, and uniquely produced for each infant, with content changing over time to promote healthy growth and development (Shenker, 2019). Breastmilk has been described as a type of personalised medicine, not only providing the infant protection from infections, but also conferring longer-term health benefits which persist into later adult life (Victora *et al*., 2016). Breastfed children are estimated to be around 30% less likely to suffer from obesity in childhood and later life (Rito *et al*., 2019), which is an important public health consideration especially as one in three children aged 6–9 years and over 50% of adults are overweight or obese in Europe (Rito *et al*., 2019). Breastfeeding also offers some protection against other non-communicable diseases such as cardiovascular disease, diabetes, cancer, and respiratory diseases, conditions which present the greatest burden of disease in Europe in the 21st century (Rollins *et al*., 2016).

Breastfeeding has also been associated with higher iQ levels (Horta *et al*., 2013; Strom *et al*., 2019), better school attendance and physical fitness rates (Tambalis *et al*., 2019), and improved job prospects and higher incomes in adult life (Victora *et al*., 2015) in low-, middle-, and high-income countries. The emotional closeness the infant has with their mother when breastfeeding responsively helps to build a strong reciprocal relationship that aids brain development, increases feelings of trust, which in turn could impact on positive mental health outcomes for mothers and their babies (Oddy *et al*., 2010; Xu *et al*., 2014; Borra *et al*., 2015; Brown, 2018).

Breastfeeding offers a number of health benefits for women including reduced risk of breast and ovarian cancer, osteoporosis, heart disease, and diabetes (Rollins *et al*., 2016). Previous detailed economic analysis reported that any investment in breastfeeding support would pay for itself in 1 year and would offer governments large savings over the lifetime of the population (Renfrew *et al*., 2012). Despite these potential health and economic benefits (Renfrew *et al*., 2012), progress to establish national breastfeeding strategies has been slow (Zakaria-Grkovic *et al*., 2020). A UK investigation carried out in England, Wales, and Scotland in 2018–2019 aimed to scale up the protection, promotion, and support for breastfeeding, using the evidence-based Yale University ‘Gear Model’, called Becoming Breastfeeding Friendly (Yale School of Public Health, 2020). This initiative led by Kent University involved multi-disciplinary teams from all three countries made recommendations on the way forward. The devolved governments in Scotland and Wales have reported on these in 2019 (Scottish Government, 2019; Eida and Kendall, 2019), but England has yet to do so.

The World Breastfeeding Trends initiative UK report (WBTi, 2016) systematically evaluated breastfeeding policies and practices using an internationally recognised tool, bringing together key organisations involved in infant and maternal health to collaborate on monitoring the WHO Global Strategy (WHO, 2003). Ten indicators were identified for evaluating policies and practices, one of which included the need to improve healthcare professionals’ training in breastfeeding. We propose that breastfeeding training needs to include how health professionals can adequately support fathers/partners as well as mothers, given the important role they play in promoting and supporting breastfeeding, as discussed in the next section.

## The importance of including fathers/partners in breastfeeding promotion and support

Research shows that fathers play an important role in promoting and supporting their partners with breastfeeding (Tohotoa *et al.,* 2011; Rempel and Rempel, 2011; Sherriff and Hall, 2011; Datta *et al*., 2012; Sherriff *et al*., 2014; Hansen *et al*., 2018). in a systematic review and meta-analysis of the effectiveness of targeting fathers for breastfeeding promotion, which included eight interventional studies from a range of countries (Australia = 1, Brazil = 1, Canada = 1, China = 1, iran = 1, italy = 1, Turkey = 2) which presented data from 1852 families, Mahesh *et al*. (2018) reported favourable results for targeting fathers in the promotion of breastfeeding. The review found breastfeeding education and promotion for fathers in the antenatal and postnatal periods improved exclusive breastfeeding rates at 6 months, decreased the probability of full formula feeding at 2 months, decreased the occurrence of breastfeeding-related problems, increased the level of support offered by the father in breastfeeding-related issues, and improved the mothers’ knowledge and attitude towards breastfeeding (Mahesh *et al*., 2018).

Despite this, fathers continue to report inadequate levels of information and support from health professionals regarding breastfeeding. Fathers’ needs relating to breastfeeding knowledge and support were identified in the recent New Dad Study (NEST) carried out in the UK (Baldwin *et al*., 2018; 2019; 2021). This was a three-part study incorporating a systematic review, a qualitative study, and a mixed-method study, aimed at exploring the mental health needs of first-time fathers.

The systematic review included 22 studies from eight countries (Australia = 3, Canada = 2, Japan = 1, Singapore = 1, Sweden = 3, Taiwan = 1, UK = 9, USA = 2), published between 1990 and 2017 found wide disparities between men’s expectations and the reality of their partner’s breastfeeding experiences (Baldwin *et al*., 2018). New fathers found breastfeeding a more difficult experience than anticipated, it was associated with increased anxiety and they felt totally unprepared to be able to support their partner to breastfeed successfully, as reflected in the following quotes from study participants:

*“i have to say that there i was not prepared at all but had a mental picture that it’s just a matter of laying the baby to the breast and it all works. When it didn’t work you stood there: aha, what the hell do we do now?”* (Baldwin *et al*., 2018, p-2132)

This often left them feeling ‘helpless’: *“Breastfeeding was what i found most difficult. i didn’t know how to help, i felt useless.”* (Baldwin *et al*., 2018, p-2132)

These findings were consistent with previous research which suggests that the attributes of positive father support in relation to breastfeeding are dependent on the father’s knowledge about breastfeeding; their attitudes to breastfeeding; their involvement in the decision-making process about breastfeeding; and their ability to provide practical and emotional support to their partner (Sherriff *et al*., 2014).

A qualitative study of 21 first-time fathers in London, England which formed the second NEST study (Baldwin *et al*., 2019), found new fathers described not knowing how to help or support their partner with breastfeeding when they were experiencing difficulties. One father stated: *“Well, i think the most difficult thing that we faced was breastfeeding, and there was a lot of information that was given and it was all, kind of, geared towards how breastfeeding is great for your child, and all of those kind of things, but it was none of the, kind of, practical tips of what to do once things start going wrong, in the sense that your child may not know how to latch. So, as a dad, what can you do to, kind of, support that?”* (Baldwin *et al*., 2019, p-7).

The third NEST study, a mixed-methods study of 52 first-time fathers, reported that one father felt that the health visitor was “*not being honest that breastfeeding can potentially be very painful*” and found this to be the least helpful aspect of their visit (Baldwin *et al*., 2021). in this third study, findings showed that men wanted more ‘realistic’ information about breastfeeding and better support for their partners, as one father stated, “*everybody said breastfeeding was easy, there was no mention that it could be hard*” (F12). in infant feeding classes, men described as being presented with “*a utopian view of how feeding would come about, you know, you take the baby and you plonk him on it, and it just works like magic*” (F19). in addition, they also reported receiving inconsistent and conflicting advice from health professionals regarding breastfeeding (Baldwin *et al*, 2021).

The findings from NEST (Baldwin *et al*., 2018; 2019; 2021) highlight the importance of providing fathers with accurate and consistent information about breastfeeding prior to the birth of their baby, and planned, ongoing postnatal support to ensure that they felt better able to support their partners. This is reflected in several earlier studies (Sherriff *et al*., 2014; Hansen *et al*., 2018; Mahesh *et al*., 2018). if fathers are to provide better support to their partners, breastfeeding is likely to be more successful, continue for longer, and women more likely to feel confident with breastfeeding (Avery and Magnus, 2011; Mannion *et al*., 2013; Sherriff *et al*., 2014; Al Namir *et al*., 2017). Additionally, timely and relevant information targeted at fathers during the perinatal period can help reduce their own anxiety, increase their problem-solving capabilities, and develop their awareness of potential breastfeeding difficulties, infant developmental milestones, and maternal postnatal depression (Sherriff *et al*., 2014).

## Health professionals’ role in providing breastfeeding information and support to fathers

Based on the last UK infant Feeding Survey, data for which were collated in 2010, over 8 out of 10 women in the UK gave up breastfeeding before they planned because they did not receive the support they needed from families, society, and professionals (McAndrew *et al*., 2012). Health professionals including midwives and health visitors are in an ideal position to enable fathers/partners to support breastfeeding, especially in the early days and weeks following birth, when the woman is establishing breastfeeding (Baldwin *et al*., 2018).

Breastfeeding programmes tailored for both parents are more effective in increasing breastfeeding rates and duration (Abbass-Dick *et al*., 2015). However, before health professionals can effectively support fathers/partners, it is important for them to understand and acknowledge the importance of the father’s/partner’s role in providing breastfeeding support to their partner, practically and emotionally (Bhairo and Elliott, 2018; Baldwin *et al*., 2021).

Effective engagement with fathers is the first step to providing breastfeeding support. Health professionals, such as midwives and health visitors have often cited their limited experience of working with fathers and their lack of training and confidence as barriers to their ability to provide adequate support (Oldfield and Carr, 2017; Whitelock, 2016; Wynter *et al*., 2021). Training for health professionals working with parents needs to incorporate training on father/partner inclusiveness and engagement. Such training has been shown to be effective in improving knowledge and attitudes and competences amongst course participants, whilst also improving organisational practices and rates of father engagement (Burgess *et al*., 2014; Burn *et al*., 2019).

Midwives and health visitors need to include and engage with fathers/partners during the routine antenatal appointments, so that they can highlight the importance of their role in the success of breastfeeding. An understanding of how babies behave instinctively after birth through skin-to-skin contact with their mothers, by latching themselves on the breasts, will enable the couple enjoy this wonderful ‘golden hour’ after birth by seeing, stroking, and connecting with their babies for the first time. Emotional support, reassurance, and encouragement offered by fathers/partners will support women’s confidence and self-efficacy (Mannion *et al*., 2013; Bhairo and Elliott, 2018).

Some parents may view formula milk as being as good as breast milk and the ‘normal’ way to feed babies. Health professionals can use antenatal contacts to explore parents’ attitudes to breastfeeding, and through the use of non-judgemental communication skills, help them to perceive breastfeeding as an achievable option, offering timely, realistic, evidence-based information on benefits. Fathers/partners need timely and appropriate information about breastfeeding during pregnancy and ongoing support postnatally to ensure they feel better able to support their partners (Sherriff *et al*., 2014; Hansen *et al*., 2018; Baldwin *et al*., 2018).

In a qualitative study which collated data from 51 health professionals in 10 focus groups with Scottish NHS staff (including health visitors and midwives), Marks and O’Connor (2015) reported that although clinicians felt positively towards breastfeeding, their role was one of informing rather than promoting and ‘moralising’ breastfeeding. ‘Moralising’ was described as associating breastfeeding with being perceived to be a ‘good’ mother (Marks and O’Connor, 2015). This is an important consideration for health professionals, and it is crucial that they offer parents accurate and realistic information about breastfeeding. This should include information about all the benefits as well as clarity about infant feeding difficulties they may face in early parenthood, and how and where to seek advice from when ‘things go wrong’ (Baldwin *et al*., 2019). Providing this level of information could help parents make an informed choice about breastfeeding.

Fathers/partners need greater support, information, and advice on the practicalities of breastfeeding, particularly in view of their frustrations about how to help their partner succeed (Wöckel *et al*., 2007; Sherriff *et al*., 2009). information and discussions with fathers/partners should be aimed at increasing their knowledge about breastfeeding; helping them develop a positive attitude towards breastfeeding; involving them in the decision-making process; and guidance on how to provide practical and emotional support to their partner.

Realistic expectations that breastfeeding takes time to establish effectively are important, but if the baby is positioning and attaching well, initial difficulties such as painful feeding, may be prevented, and likely to be resolved. Parents’ understanding of new-born babies’ behaviour will help them realise that ‘fussiness’ at certain times of the day may have nothing to do with how they are fed. For more complex problems, health professionals can signpost fathers/partners to access help from local infant feeding teams, the National Breastfeeding Helpline, third sector breastfeeding counsellors or lactation consultants. An integrated approach with other infant feeding agencies and peer support services will ensure parents receive the optimum support they need to achieve their goals.

it is crucial that fathers/partners are offered guidance on how to support their partner in ways other than direct infant feeding such as giving their partner time to rest, making them food and drink, offering regular praise, reassurance, and encouragement, as these may not be obvious interventions (Sherriff *et al*., 2009). involving fathers/partners in the breastfeeding decision making process as early as possible has the potential to make them breastfeeding advocates, where they can protect and defend parenting decisions against negative or unhelpful interference, for example from their extended family who may encourage artificial milk feeding or undermine their partner’s efforts (Pontes *et al*., 2009; Tohota *et al*., 2011; Sherriff *et al*., 2014). Preparing and supporting fathers appropriately could also increase their self-confidence and self-efficacy (Datta *et al*., 2012). This has the potential to increase breastfeeding rates and duration, contributing to better outcomes for babies, mothers, and the wider public health agenda.

## Recommendations for supporting fathers/partners

Based on the evidence presented in this article, we propose the following strategies that health professionals could use to involve and support fathers/partners with breastfeeding:

- involve and engage with fathers/partners in the decision-making process about infant feeding in the antenatal period.
- Provide fathers/partners with appropriate information about breastfeeding prior to the birth of their baby, to include the short-term and long-term benefits.
- inform fathers about the importance of their role in supporting their partner with breastfeeding.
- Have ‘realistic’ discussions about breastfeeding, informing parents that breastfeeding is a skill that may take time to get the hang off. include ‘frank discussions’ about the difficulties they may face in early parenthood (such as sleepless nights, exhaustion, relationship changes etc.) and what to do when ‘things go wrong’
- Enquire about and explore fathers’/partners’ views about breastfeeding and any anxieties or uncertainties they may have.
- if fathers/partners are worried about missing out, educate them about other ways of getting involved with their baby, such as skin-to-skin contact, playing, bathing, changing nappies, talking, using a sling etc.
- Provide fathers/partners with information about the practical support they can offer their partners to support breastfeeding, such as helping with household duties, giving them a massage, allowing them to rest, making food and drinks for them, restricting visitors, and finding additional sources of support for them if necessary.
- Provide fathers/partners with information about the emotional support they can offer their partners during breastfeeding, such as reassurance and encouragement.
- Ensure fathers/partners (as well as mothers) are aware of local and national breastfeeding support services and how to access them. These should include online resources, telephone helplines, and support groups.
- Continue to engage with and provide ongoing breastfeeding support to fathers/partners following the birth to ensure that they are kept well informed and can continue to support their partners.
- Encourage fathers/partners to seek specialist support if their partner is experiencing any breastfeeding difficulties.

it is important that health professionals meet the needs of fathers/partners with relation to breastfeeding. Regular feedback from parents will enable the collection of such data to evaluate the support provided and make the necessary adjustments when needed. Any steps taken to include, involve, and advise fathers/partners should also be documented in the family records to provide an audit trail and allow for further evaluation to be undertaken. Local and national resources, policies, and guidelines for fathers need to be designed acknowledging the fathers’ role (Bhairo and Elliott, 2018).

## Conclusion/summary

From the evidence, breastfeeding offers many health benefits for the maternal–infant dyad and increasing breastfeeding rates globally could have a positive impact on global public health. As fathers/partners play an important role in promoting and supporting women with breastfeeding, it is crucial that health professionals provide adequate information and support to them too. The contacts that midwives and health visitors have with women in the antenatal and postnatal periods should include their partners whenever possible. Discussions around breastfeeding during these contacts could increase partner’s knowledge, confidence, and expectations, enabling them to support their partners in an informed way. informing both parents about the practical aspects of breastfeeding is just as important and should include providing them with details of how to access further professional support if needed. While it is recognised that only providing fathers/partners with information about breastfeeding may not be enough to change their attitudes or behaviour towards breastfeeding, it could nonetheless act as a starting point to increasing father engagement and participation in breastfeeding. Midwives and health visitors are in ideal positions to offer mothers, fathers/supportive partners timely and relevant breastfeeding information and support throughout the perinatal period. Further research is needed to explore what influences fathers’/partners’ attitudes to breastfeeding and how their needs relating to infant feeding could be best addressed by health professionals. The creation of guidance to support fathers/partners with breastfeeding that includes fathers from diverse backgrounds is likely to make it more acceptable to men.
